# Trimethoprim Use prior to Pregnancy and the Risk of Congenital Malformation: A Register-Based Nationwide Cohort Study

**DOI:** 10.1155/2013/364526

**Published:** 2013-02-14

**Authors:** Jon Trærup Andersen, Morten Petersen, Espen Jimenez-Solem, Jeppe Nørgaard Rasmussen, Nadia Lyhne Andersen, Shoaib Afzal, Kasper Broedbaek, Brian Rafn Hjelvang, Lars Køber, Christian Torp-Pedersen, Henrik Enghusen Poulsen

**Affiliations:** ^1^Laboratory of Clinical Pharmacology, Copenhagen University Hospital Rigshospitalet, Blegdamsvej 9, 2100 Copenhagen, Denmark; ^2^Department of Clinical Pharmacology, Bispebjerg Hospital, Bispebjerg Bakke 23, 2400 Copenhagen, Denmark; ^3^Center for Healthy Ageing, Faculty of Health Sciences, University of Copenhagen, Blegdamsvej 3, 2200 Copenhagen, Denmark; ^4^Mental Health Centre Copenhagen, Bispebjerg Bakke 23, 2400 Copenhagen, Denmark; ^5^The Heart Centre, Copenhagen University Hospital Rigshospitalet, Blegdamsvej 9, 2100 Copenhagen, Denmark; ^6^Department of Cardiology, Gentofte University Hospital, Niels Andersens Vej 65, 2900 Hellerup, Denmark; ^7^Faculty of Health Sciences, University of Copenhagen, Blegdamsvej 3, 2200 Copenhagen, Denmark

## Abstract

*Objectives*. The aim of the study was to investigate whether the use of the antifolate antibiotic trimethoprim during the 12 weeks before conception was associated with congenital malformations. *Methods*. We conducted a nationwide register-based cohort study including all Danish women giving birth from 1997 to 2004. All women with at least one prescription of trimethoprim dispensed during the 12 weeks before conception were identified. *Results*. There was a doubling of congenital malformations in offspring to women exposed to trimethoprim in the 12 weeks before conception. The adjusted odds ratio (OR) of major congenital malformation was 1.87, 95% confidence interval (CI) 1.25–2.81. There was a significant increase in major malformations of the heart (OR = 2.49; 1.18–5.26) and limbs (OR = 2.18; 1.13–4.23). *Conclusions*. In this study, we found an association between exposure to trimethoprim during the 12 weeks before conception and an increased risk of heart and limb defects.

## 1. Introduction 

The antibiotic trimethoprim is an inhibitor of dihydrofolate reductase (DHFR), which converts folate to more active components [1]. This folate antagonism of trimethoprim has been associated with several types of congenital malformations and miscarriage when given during pregnancy [2, 3]. This effect is almost entirely abolished by concomitant use of vitamin supplements containing folic acids [2]. The use of multivitamins including folic acid has been associated with a reduced risk of having offspring with cardiovascular [4–6], urinary tract [5, 7], oral cleft [6, 7], limb [7, 8], and neural tube defects [6, 9, 10]. This further strengthens the evidence of a direct association between DHFR inhibition and malformation. Theoretically, DHFR inhibitors can result in folate deficiency that lasts into the pregnancy period. A risk of congenital malformations occasioned by DHFR inhibitor use prior to pregnancy could have serious consequences. Trimethoprim is widely used throughout the world to treat urinary tract, respiratory tract, and gastrointestinal infections and is used as daily prophylaxis in HIV infection and AIDS treatment in Africa [11]. Due to its common use, large numbers of fertile women are exposed to the drug before becoming pregnant.

We therefore tested the hypothesis that trimethoprim use during the months prior to pregnancy increases the risk of congenital malformations. To this end, we performed a nationwide study in which administrative registers of trimethoprim use were coupled with registrations of birth and congenital malformations.

## 2. Methods

### 2.1. Study Population

Using the Danish Fertility Database, [12] we identified all conceptions in Denmark between January 1, 1997, and December 31, 2004, that resulted in live births (*n* = 521, 611). Follow-up data was available to December 31 2005. From these, 344 were excluded due to coding error. Information about maternal drug use before and during pregnancy was obtained from the Register of Medicinal Product Statistics (The National Prescription Register), and children with congenital malformations were identified through the National Hospital Register.

The Danish Fertility Database consists of individual-level data about the mother and the father, including a unique identification number, age, previous births, and abortions, as well as birth weight and length, death and cause of death, sex, and gestational age of the children [12]. The time of conception is based on ultrasound results or information about the date of the last menstruation. The Register of Medicinal Product Statistics contains individual-level data about all prescribed drugs dispensed at a pharmacy in Denmark since 1995 [13]. The register has no information about over-the-counter drugs or the indication of treatment. The National Hospital Register contains information on all hospitalizations in the country, including admittance data and discharge diagnosis [14].

Primary outcome was predefined as all congenital malformations (ICD-10 Q0.00–Q99.9). The secondary outcome was the subgroup of malformations. All major and minor malformations and malformation groupings are those of the European Surveillance of Congenital Anomalies (EUROCAT) classification system guide 1.3 [15]. Exposure was defined as at least one dispensed prescription of a systemic drug containing trimethoprim (Anatomical Therapeutic Chemical Classification System (ATC) J01EA01 and J01EE01). The exposure period was predefined as the 12 weeks prior to conception since this is the previously estimated risk period of methotrexate [16].

Several different analyses were done. Offspring of mothers with no dispensed trimethoprim prescriptions in the 12 weeks before conception were used as the primary reference group as well as offspring of mothers with no dispensed prescriptions in the 12 weeks before pregnancy and during pregnancy. To avoid confounding by indication, we used the offspring of mothers with one or more dispensed prescriptions of the mecillinam, a penicillin used to treat urinary tract infections, during the 12 weeks before pregnancy as a reference group. Furthermore, we compared the results with the number of children diagnosed with a congenital malformation in women exposed to trimethoprim solely in weeks 13–24 and in weeks 25–36 before conception.

Comorbidity of mothers was assessed through the National Hospital Register on the basis of diagnoses made between 1995 and 2005. This was done to assess whether women treated with trimethoprim were more likely to be diagnosed with a teratogenic infection or conditions potentially harmful to the fetus. Diagnoses of infection, alcoholism, amniocentesis, and folate deficiency were included if they were given during pregnancy, whereas chronic conditions were included if the diagnoses were given before or during pregnancy. All prescribed drugs dispensed before and during pregnancy were classified according to the US Food and Drug Administration's *Pregnancy Risk Factor* Category X [17] (drugs with evidence of human foetal risk) in order to identify the usage of these drugs in the cohort. The use of folic acid was defined as redemption of a prescription of folic acid in the period from 84 days before conception to the end of the first trimester.

### 2.2. Statistical Analysis

All data management and analyses were performed using SAS software, version 9.1 (SAS Institute Inc., Cary, NC, USA). Three logistic regression models were used on dichotomous outcomes to estimate the odds ratios of congenital malformation. Model one was unadjusted; model two was adjusted for maternal age (as a continuous variable) and parity (as a continuous variable). We adjusted model three for maternal age (as a continuous variable), parity (as a continuous variable), income (four categories), education (four categories), usage of X-classified drugs (dichotomous variable), calendar time (1997-1998, 1999-2000, 2001-2002, 2003-2004), teratogenic infections (dichotomous variable, if the mother had at least one diagnosis of either syphilis, toxoplasmosis, rubella viruses, varicella virus, parvovirus B-19, cytomegalovirus, herpes simplex virus 1 and 2, or Venezuelan equine encephalitis virus during pregnancy), and folate deficiency, myasthenia gravis, virilizing tumors, diabetes mellitus, alcoholism, amniocentesis, and Sjogren's syndrome (all as dichotomous variables). These variables had less than 0.5% missing values except for education (2.5% in the trimethoprim exposed group and 2.6% in the unexposed group (*P* = 0.93)).

For all analyses, a two-sided value of *P* < 0.05 was considered statistically significant and odds ratios are presented with 95% confidence intervals.

### 2.3. Ethics

All data were linked in computers held by Statistics Denmark and were made available with encrypted personal information [18]. This ensured that no individuals could be identified. Ethical permission is not required in Denmark for retrospective register studies. The Danish Data Protection Agency approved the study (no. 2008-41-2517). We report our findings according to strengthening the reporting of observational studies in epidemiology (STROBE) [19].

## 3. Results

We identified 521,267 births during the study period. A total of 402 children were born to 395 women exposed to a drug containing trimethoprim (trimethoprim or sulfamethoxazole plus trimethoprim) during the 12 weeks before conception (Table 1). Women exposed to trimethoprim were more likely to be younger (*P* < 0.001) and have lower education (*P* = 0.02) and lower household income (*P* = 0.02) in comparison to nonexposed women (Table 1).

Of the children born to an exposed mother, 25 (6.2%) were diagnosed with a major congenital malformation compared with 17,465 (3.4%) among those born to unexposed mothers. Among the exposed women, 40 (10.0%) children were born with a diagnosed congenital malformation (including minor malformations) compared with 27,173 (5.2%) born to unexposed mothers. 

The odds ratio (OR) for the occurrence of a major congenital malformation after exposure to trimethoprim during the 12 weeks before pregnancy (model 1) was 1.91 (95% confidence interval 1.28–2.87) compared to unexposed women. Adjusting for age and parity (model 2), the OR was 1.91 (1.27–2.86). Furthermore, we included usage of X-classified drugs, teratogenic infections, folate deficiency, myasthenia gravis, virilizing tumors, diabetes mellitus, alcoholism, amniocentesis, Sjogren's syndrome, education, calendar time, income, and use of folic acid in addition to age and parity (model 3) in the regression model without any influence on the result (OR = 1.87; 1.24–2.80) (Table 2).

**Figure 1:**
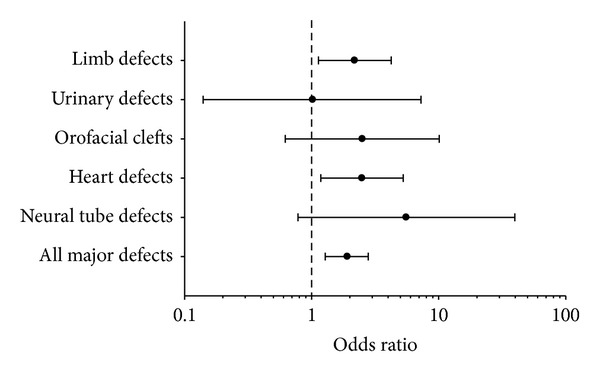
Incidence of types of major congenital malformation in children of women exposed to trimethoprim before conception as compared to children of women unexposed to trimethoprim before conception. Only types of malformations reduced by folic acid supplementation before or during pregnancy are included in the figure. Odds ratio (95% CI).

Of the children with major malformations in the trimethoprim group, seven had a malformation of the heart (OR = 2.49; 1.18–5.26) and nine had a congenital malformation of the limbs (OR = 2.18; 1.13–4.23) (Table 3, Figure 1). No other subgroupings of malformations were significantly increased (Table 3).

### 3.1. Other Analyses

In Denmark, trimethoprim exists in packages for 7.5 defined daily doses (DDD), 25 DDD, and in combination with sulfamethoxazole as 7.5 DDD. The 7.5 DDD trimethoprim package is the most common, accounting for 77% of exposures. To confirm that the findings relate to preconception exposure, we performed a logistic regression analysis that only included women redeeming prescriptions of 7.5 DDD trimethoprim and only 14–84 days before conception; this ensured a minimum of one-week washout period before conception. In this analysis, the odds ratio was 1.81 (1.11–2.96) compared to unexposed women in the same period. 

Despite the very low statistical power, we analysed the effect of the three treatment regimes. Women with a prescription of 7.5 DDD trimethoprim (*n* = 285) were more likely to give birth to children with major congenital malformations (OR = 2.06; 1.29–3.28). Women redeeming prescriptions for 25 DDD trimethoprim (*n* = 45) or trimethoprim and sulfamethoxazole in combination (*n* = 75) had a higher risk of having a child with a major congenital malformation (OR = 2.06; 0.64–6.64 and OR = 1.62; 0.59–4.44, resp.). 

There was no difference in the risk estimate when adjusting for age of offspring at the time of diagnosis and no association between the year of conception and the number of children born with a major malformation (*P* = 0.15).

A secondary reference group of women exposed to mecillinam in the 12 weeks before conception was used in a direct comparison with the women exposed to trimethoprim in the same period. For this calculation, there was still an increased risk of having a child with a congenital malformation when exposed to trimethoprim compared to being exposed to mecillinam in the 12 weeks before conception (OR = 1.78; 1.15–2.77). Of the children with major malformations, seven (28.0%) had multiple malformations in the exposed group compared with 4799 (27.5%) in the unexposed group (OR = 1.03; 0.43–2.46).

There was no difference in the incidence of major congenital malformations in children born to women dispensing trimethoprim solely in weeks 13–24 (OR = 1.20; 0.73–1.98) or weeks 25–36 (OR = 1.13; 0.68–1.90) before conception.

A post hoc analysis regarding the women exposed to trimethoprim within the 50 days before conception was made and the odds ratio was only minimally different compared to the 12-week period prior to conception (OR = 2.06; 1.26–3.37). Two women in the trimethoprim group were exposed to a drug classified X during their pregnancy, but neither of them had offspring with congenital malformations.

## 4. Discussion

In the present study, we found a doubling in the prevalence of major congenital malformations in offspring of women exposed to trimethoprim during the 12 weeks before conception. According to the EUROCAT subgrouping, there was an increased prevalence of heart and limb defects. 

We considered several sources of possible bias. Infections may be teratogenic, and in Denmark, for younger women, trimethoprim is almost exclusively used to treat urinary tract infections. To rule out the possibility that these infections are the cause of malformations, we compared our results with those from women receiving mecillinam, in Denmark, a widely used penicillin primarily to treat urinary tract infections. 

The result of this analysis (Table 2) shows that the use during the same preconceptional period of mecillinam is not associated with a similarly increased risk of congenital malformations. This indicates that confounding due to urinary tract infections is unlikely. The trimethoprim-exposed and -unexposed groups are not comparable with respect to age, education, and income. On average, the women exposed to trimethoprim were 1.2 years younger and had a lower income and educational status. Nevertheless, additional logistic regression analyses taking these differences into account did not change the results substantively. To exclude the importance of other characteristics of women using trimethoprim, we compared the result with dispensing of trimethoprim much earlier than the pregnancy period but found no increased risk with dispensing 13–24 weeks and 25–36 weeks before pregnancy. 

Multivitamin supplementation containing folic acid used prior to and during early pregnancy has been associated with a reduced risk of having offspring with malformations, amongst others, of the limbs and heart [4–8]. Furthermore, heart and limb defects are typically associated with genetic factors and exposure to thalidomide, rubella virus, and alcohol during pregnancy. Since we found an increase in these malformations only, this could indicate that folate deficiency could explain the observed association between malformations and preconceptional dispensing of trimethoprim. We also found a higher odds ratio of neural tube defects and orofacial clefts, which are malformations that are also reduced by the use of folic acid (Figure 1). However, this was statistically insignificant and it was based on a very small number of cases in our study. Overall, we do not have sufficient exposed cases to conduct valid analyses for all EUROCAT subgroups.

The mechanism of trimethoprim as a folic acid antagonist could explain the association found, and the previous studies have shown that trimethoprim can reduce serum folate levels [20, 26–28]. We find it possible that women with a low folate level before commencing treatment could be especially vulnerable to trimethoprim and thereby be exposed to folate deficiency that affects the foetus during the first trimester. Trimethoprim works by inhibiting dihydrofolate reductase, with a similar mode of action to that of the recognized teratogenic drug methotrexate, which, like other cytotoxic agents, is suspected of inducing malformations when given during the months leading up to pregnancy. Even though no studies have been able to show an association between preconceptional methotrexate and congenital malformations [29, 30], it is recommended that safe contraception be used during and for at least 3–6 months after treatment with methotrexate [16, 31, 32]. Methotrexate is thought to be retained for several weeks in the kidneys and for months in the liver, thereby affecting the fertilized ova [16], but we are unaware of any equivalent reports of the retention of trimethoprim.

**Figure 2:**
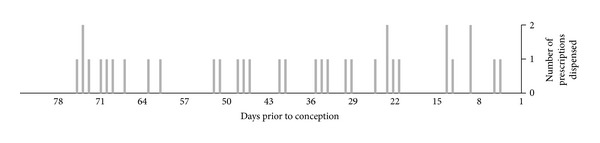
Dispensing day of trimethoprim in the 12-week period prior to conception for mothers of children with major and minor malformations. Only women who dispensed one prescription of trimethoprim during the period and had no prescriptions for 85–336 days prior to pregnancy and 90 days after conception are included in the figure.

In the present study, the period of interest was predefined as being 12 weeks but, in truth, we do not know how long the vulnerable period prior to pregnancy is. One previously published study has indicated that treatment with trimethoprim may lower plasma folate for up to 50 days [20]. We therefore made a post hoc analysis of the 50 days period before pregnancy and found an increased risk of having a child with a malformation (OR = 2.06; 1.26–3.37) compared to trimethoprim use in the 84 days period prior to pregnancy (Figure 2). However, we found no increase in the number of malformations when using trimethoprim 13–24 and 25–36 weeks prior to conception. 

If the association between trimethoprim and malformations was due to induced folate deficiency, we would expect to find fewer children born with malformations in the study population in the years after introduction of an official folic acid supplementation policy. Official recommendations regarding folic acid supplementations were introduced in Denmark in 1997. At that time, it was estimated that only 5% of the Danish women received the recommended daily dosage of folate [21]. In 2004, at the end of our study period, compliance was very low, such that only 22% of women with a planned pregnancy followed the official recommendations [22]. In the present study, we did not find any association between the numbers of malformations and the year of conception. This could very well be because of the poor compliance in Danish women during the study period.

Prescription data and malformation data have been studied before and found to be accurately recorded [13, 23, 24]. Pharmacies are required to register prescriptions and this activity is coupled with the reimbursement of expenses from the state, which ensures highly accurate prescription data [13]. Completeness has previously been estimated to be 97.5% [23]. The quality of the diagnoses of congenital malformations has been validated and found to have a predictive value of 88.2% for having a congenital malformation, with a completeness of 89.9% [24]. More than 99.5% of the births in Denmark since 1978 are registered in the Danish Fertility Database [12].

The strengths of this study are the large number of cases and its nationwide coverage, including all women giving birth in Denmark during the study period. This ensures very high completeness of the data independent of age, race, and social, educational, and economic statuses, which thereby minimizes the selection bias. Furthermore, the study only includes women who obtained and paid for the medication at the pharmacy. 

The main limitation of the study is a potential existence of unaccounted confounding. Women using trimethoprim preconceptionally may differ in important characteristics from women who do not receive the treatment. Such characteristic could be causally related to the pregnancy outcome and confound partially or entirely the observed association. Another limitation is the lack of information concerning dosage and compliance. Even though we know about package and tablet size, we have no precise information about the dose prescribed. For this reasons we have restricted the analysis to treatment or no treatment. Furthermore, we do not know about the indication for treatment, for which reason we cannot completely rule out the possibility that the results are confounded by indication. To meet and thereby minimize these limitations, we have adjusted the analyses for teratogenic conditions, age, parity, and so forth and used an alternative reference group of mecillinam users. Low compliance would bias the study towards a lack of effect and the estimated risk may therefore be underestimated. If the treatment was taken later, then the effect might be overestimated because of possible postconception administration. This would have only a minor effect since trimethoprim is mainly used for acute infections in Denmark. Furthermore, it has previously been estimated that the majority of prescription drugs redeemed are taken [25]. 

In model 3 we tried to adjust the analyses for conditions potentially harmful to the fetus. These conditions include medical conditions such as some autoimmune diseases and diabetes mellitus, infections during pregnancy potential harmful to the fetus, and diagnoses of folate deficiency. This information was taken from the National Hospital Register and only included women actually diagnosed with the conditions. If women had any of the conditions but were not diagnosed at a hospital, the information was not available to us. This is a limitation and could potentially confound our adjusted result. Furthermore, we adjusted for use of folic acid. This information is based on redeemed prescriptions on folic acid and is very deficient since the majority of folic acid and multivitamin containing folic acid used in Denmark are sold over the counter.

Trimethoprim is used by millions of fertile women worldwide. The World Health Organization (WHO) recommends that adults with symptomatic HIV or AIDS should use life-long daily prophylaxis with co-trimoxazole (trimethoprim and sulfamethoxazole) [11]. This recommendation affects more than 12 million fertile women in sub-Saharan Africa who are estimated to live with HIV infection or AIDS [33, 34]. Furthermore, worldwide, huge numbers of women use shorter courses of trimethoprim to treat urinary infections. 

The scale of the problem could be even larger in some countries. In the United Kingdom, the Medicines and Healthcare Products Regulatory Agency has been discussing whether trimethoprim should be reclassified for over-the-counter availability [35]. This could make trimethoprim the primary medication for urinary tract infections and thereby increase its use and the exposure of fertile women. A very large number of pregnant women could be exposed prior to pregnancy as a result of such a reclassification, especially as only 50% of fertile women are estimated to plan their pregnancy [36].

Viewed together, although we find the results of the present study biologically plausible, it is the first time this hypothesis has been tested. It is important to test the hypothesis in other studies. Furthermore, it is important to keep in mind that prophylaxis with trimethoprim-sulfamethoxazole in people living with HIV or AIDS in sub-Saharan Africa has been shown to reduce morbidity and mortality [37–39]. A more general implication of our study concerns pharmaceutical treatment prior to pregnancy. If trimethoprim can be retained or is able to induce lasting folate deficiency, then the use of other drugs may also bear a risk. Our study identifies the need for further research into the risk of teratogenicity in the months leading up to pregnancy.

In conclusion, we have found an association of maternal exposure to trimethoprim during the 12 weeks before conception and a doubling of the rate of congenital malformations in the subsequent offspring. This calls for further investigation, both epidemiological and in vivo animal studies. 

## Figures and Tables

**Table 1:** Study population characteristics.

	Use of trimethoprim in the 12-week period prior to pregnancy	No use of trimethoprim in the 12-week period prior to pregnancy
	*n* = 402	*n* = 520, 865
Age (year)	28.5	29.7
Parity (mean)	1.80	1.85
Household income—number (%)		
<$50,000	76 (19)	80,899 (16)
$50,000–$100,000	194 (48)	241,907 (46)
$100,000–$150,000	110 (27)	150,163 (29)
>$150,000	22 (5)	48,240 (9)
Education—number (%)		
Low	168 (42)	182,072 (35)
Medium	123 (31)	165,132 (32)
Long	98 (24)	149,324 (29)
No information available	10 (2)	13,333 (3)
Comorbidity^b^—number (%)		
Teratogenic infectious diseases^c^	1^a^ (0.3)	133 (0.0)
Folate deficiency	0 (0)	39 (0.0)
Myasthenia gravis	0 (0)	38 (0.0)
Virilizing tumors	0 (0)	84 (0.0)
Diabetes Mellitus	4 (1)	6258 (1)
Alcoholism	0 (0)	111 (0.0)
Amniocentesis	1^a^ (0.3)	5381 (1.0)
Sjogren's syndrome	0 (0)	38 (0.0)
HIV/AIDS	0 (0)	60 (0.0)
*Pneumocystis carinii* pneumonia	0 (0)	2 (0.0)
Previous organ transplantation	1^a^ (0.0)	49 (0.0)
Use of folic acid—number (%)	9 (2)	4296 (1)

^a^None had offspring with a congenital malformation. ^b^All information on comorbidity were based on diagnoses from the National Hospital Register. ^c^Syphilis, toxoplasmosis, rubella virus, varicella virus, parvovirus B-19, cytomegalovirus, herpes simplex viruses 1 and 2, and Venezuelan equine encephalitis virus.

**Table 2:** Risk of congenital malformations associated with trimethoprim use during the 12-week period prior to conception.

Reference group	All unexposed children born 1997–2004	All unexposed children born 1997–2004 adjusted^a^	Children born 1997–2004 by mothers with one or more prescription of mecillinam prior to pregnancy	Children born 1997–2004 by mothers with no usage of prescription drugs prior to and during pregnancy
	*n* = 520, 865	*n* = 520, 865	*n* = 3510	*n* = 91, 332
Major congenital malformations or (CI 95%)	1.91 (1.28–2.87)	1.87 (1.25–2.80)	1.78 (1.15–2.77)	2.05 (1.37–3.08)
All congenital malformations or (CI 95%)	2.01 (1.45–2.78)	1.96 (1.41–2.72)	1.90 (1.33–2.72)	2.18 (1.57–3.03)

^a^Adjusted for age, parity, usage of X-classified drugs, teratogenic infections, folate deficiency, myasthenia gravis, diabetes mellitus, alcoholism, amniocentesis, Sjogren's syndrome, virilizing tumors, education, and income.

**Table 3:** Types of major congenital malformations observed.

Type of major malformation	Number of malformations		Odds ratio
	Exposed	Unexposed	(95% CI)
Congenital malformations of the nervous system	2 (0.5%)	686 (0.1%)	3.79 (0.94–15.25)
Neural tube defects	1 (0.3%)	234 (0.0%)	5.55 (0.78–39.68)
Congenital malformations of the eye	0 (0%)	586 (0.1%)	
Congenital malformations of the ear, face, and neck	0 (0%)	274 (0.1%)	
Congenital malformations of the heart	7 (1.7%)	3682 (0.7%)	2.49 (1.18–5.26)
Congenital malformations of the respiratory system	0 (0%)	469 (0.1%)	
Orofacial clefts	2 (0.5%)	1036 (0.2%)	2.51 (0.62–10.09)
Congenital malformations of the digestive system	2 (0.5%)	993 (0.2%)	2.62 (0.65–10.52)
Abdominal wall defects	0 (0%)	145 (0.0%)	
Congenital malformations of the external genital organs	1 (0.3%)	1442 (0.3%)	0.82 (0.13–6.40)
Congenital malformations of the internal urinary system	1 (0.3%)	1273 (0.2%)	1.02 (0.14–7.25)
Congenital malformations of the limbs	9 (2.2%)	5416 (1.0%)	2.18 (1.13–4.23)
Congenital malformations of the musculoskeletal system	1 (0.3%)	798 (0.2%)	1.62 (0.22–11.60)
Other malformations	1 (0.3%)	662 (0.1%)	1.96 (0.28–13.98)
Teratogenic syndromes with malformations	0 (0%)	38 (0.0%)	
Genetic syndromes and microdeletions	1 (0.3%)	315 (0.1%)	4.62 (0.58–29.44)
Chromosomal abnormalities	1 (0.3%)	653 (0.1%)	1.99 (0.28–14.17)

All major congenital malformations	25 (6.2%)	17,465 (3.4%)	1.91 (1.28–2.87)
All minor congenital malformations	18 (4.5%)	11,600 (2.2%)	2.06 (1.28–3.31)
All congenital malformations	40 (10.0%)	27,173 (5.2%)	2.01 (1.45–2.78)

Major congenital malformations among children of mothers exposed to trimethoprim in the 12-week period prior to conception and among unexposed according to the EUROCAT classification system.
